# Chlortetracycline, a Novel Arf Inhibitor That Decreases the Arf6-Dependent Invasive Properties of Breast Cancer Cells

**DOI:** 10.3390/molecules26040969

**Published:** 2021-02-12

**Authors:** Eric Macia, Monserrat Vazquez-Rojas, Alessia Robiolo, Racha Fayad, Sophie Abélanet, Isabelle Mus-Veteau, Fabien Fontaine-Vive, Mohamed Mehiri, Frédéric Luton, Michel Franco

**Affiliations:** 1 Institut de Pharmacologie Moléculaire et Cellulaire (IPMC), UMR 7275 CNRS-Université Côte d’Azur, 660, Route des Lucioles, 06560 Valbonne, France; macia@ipmc.cnrs.fr (E.M.); monserrat.vr@gmail.com (M.V.-R.); alessia.rbl@gmail.com (A.R.); racha.fayad1@gmail.com (R.F.); abelanet@ipmc.cnrs.fr (S.A.); musveteau@ipmc.cnrs.fr (I.M.-V.); luton@ipmc.cnrs.fr (F.L.); 2 Institut de Chimie de Nice (ICN), UMR 7272 CNRS-Université Côte d’Azur, 28, Avenue de Valrose, CEDEX 2, 06108 Nice, France; fabien.fontaine-vive@univ-cotedazur.fr (F.F.-V.); Mohamed.Mehiri@unice.fr (M.M.)

**Keywords:** small G protein, inhibitor, cell invasion, Arf6, breast cancer, chlortetracycline

## Abstract

Breast cancer is a major disease for women worldwide, where mortality is associated with tumour cell dissemination to distant organs. While the number of efficient anticancer therapies increased in the past 20 years, treatments targeting the invasive properties of metastatic tumour cells are still awaited. Various studies analysing invasive breast cancer cell lines have demonstrated that Arf6 is an important player of the migratory and invasive processes. These observations make Arf6 and its regulators potential therapeutic targets. As of today, no drug effective against Arf6 has been identified, with one explanation being that the activation of Arf6 is dependent on the presence of lipid membranes that are rarely included in drug screening. To overcome this issue we have set up a fluorescence-based high throughput screening that follows overtime the activation of Arf6 at the surface of lipid membranes. Using this unique screening assay, we isolated several compounds that affect Arf6 activation, among which the antibiotic chlortetracycline (CTC) appeared to be the most promising. In this report, we describe CTC in vitro biochemical characterization and show that it blocks both the Arf6-stimulated collective migration and cell invasion in a 3D collagen I gel of the invasive breast cancer cell line MDA-MB-231. Thus, CTC appears as a promising hit to target deadly metastatic dissemination and a powerful tool to unravel the molecular mechanisms of Arf6-mediated invasive processes.

## 1. Introduction

The ADP-Ribosylation Factor (Arf) subfamily belongs to the Ras superfamily of small GTP binding proteins. They play a crucial role in vesicular transport both in the secretory and endocytic pathways. Like all the small G-proteins, Arfs cycle between inactive GDP-bound and active GTP-bound conformations. In cells, the activation of Arfs is triggered by a GDP/GTP nucleotide exchange catalysed by the Sec7-domain containing exchange factors family. The reversion to the inactive conformation results from the hydrolysis of the third phosphate of the GTP stimulated by the Arf-GAP (GTPase Activating Proteins) family. The Arf subfamily includes five isoforms in humans; two of which, Arf1 and Arf6, have been particularly well studied. Arf1 is known to promote the formation of membrane vesicles by recruiting coat proteins to assume intra Golgi apparatus and Golgi-ER transport, whereas Arf6 is known to coordinate endocytic recycling and actin cytoskeleton reorganization at the plasma membrane.

As the intracellular transport and the regulation of the actin cytoskeleton are involved in different key cellular patho-physiological processes, it is not surprising that Arf activity is found to be associated with cancer progression. Notably, the Arf6 isoform has been described as an important mediator of cancer cell migration and invasion. A strong correlation between high levels of Arf6 expression and invasive properties of breast cancer cells has been established [1,2,3,4]. Conversely, Arf6 silencing inhibits the invasive capacity of melanoma, glioma and breast cancer cells [1,5,6]. A molecular cascade consisting of the Arf-GEF GEP100, Arf6 and the Arf-GAP AMAP1 promotes tumor invasion and metastasis in breast cancer in response to EGF receptor activation [3]. This pathway could also be involved in other cancers such as head and neck squamous cell carcinoma and lung adenocarcinoma. (reviewed in [7]). A triple positive expression of p-EGFR, GEP100 and Arf6 is associated with an increased risk of death in patients with primary lung adenocarcinoma [8]. By recruiting metalloproteases (MT1-MMP), actin binding proteins (cortactin, Arp2/3), motor proteins (dynein-dynactin, kinesin-1) and adhesion molecules (paxillin, cortactin), Arf6 could control cell migration and invadopodia formation [9].

Arf1 has also been implicated in cancer biology. Arf1 expression appears to control the proliferation of breast cancer and myeloma cells, and cell growth in prostate cancer [10,11,12,13]. In addition, Arf1 was found to be up regulated in ovarian tumors [14]. The molecular connection between Arf1 and cell proliferation is not clearly established but could be mediated through the activation of the FAK and Src kinases [12]. In summary, Arf6 would control cell motility, invasion and metastasis, whereas Arf1 would be involved in tumor cell growth.

Thus, Arf inhibitors might be valuable tools as anti-tumor chemotherapy. The fungal metabolite Brefeldin A (BFA) was the first Arf inhibitor described. It blocks the anterograde ER/Golgi transport leading to the collapse of the Golgi apparatus by inhibiting the activation of Arf1 by the exchange factor GBF1. BFA is an uncompetitive inhibitor that targets the Arf/GEF complex intermediate of the nucleotide exchange reaction. BFA was shown to induce autophagy in vitro and to act synergistically with antimitotic drugs to inhibit colorectal growth [15].

Recently, several Arf inhibitors have been isolated using different strategies [16,17]. However, to date, no Arf6 inhibitors blocking the invasion of tumour cells have been reported.

Here, by screening the Prestwick chemical library for an Arf6 inhibitor, we focused on the tetracycline antibiotic family. Tetracycline is a natural broad-spectrum antibiotic that comes from *Streptomyces* strains. Chlortetracycline (CTC, trade name Aureomycin) was the first of the tetracycline family to be discovered in 1945. Since then, many tetracyclin derivatives have been developed to form a large family. Most of them prevent bacterial protein translation by inhibiting the aminoacyl-tRNA to the ribosomal A site. A few tetracycline family members have also been shown to bind to the elongation factor EF-Tu, a bacterial G protein involved in the delivery of aa-tRNA to the ribosome. However, structural and biochemical analyses have demonstrated that interaction with tetracyclin does not affect the function of EF-Tu [18]. Here, we report the identification and the characterization of chlortetracycline (CTC) as a potent chemical inhibitor of Arf6 activity and Arf6-dependent cancer cell invasion in vitro.

## 2. Results

### 2.1. CTC Inhibits Both GEF-Stimulated and Spontaneous Activation of Arf6 In Vitro

**Figure 1:**
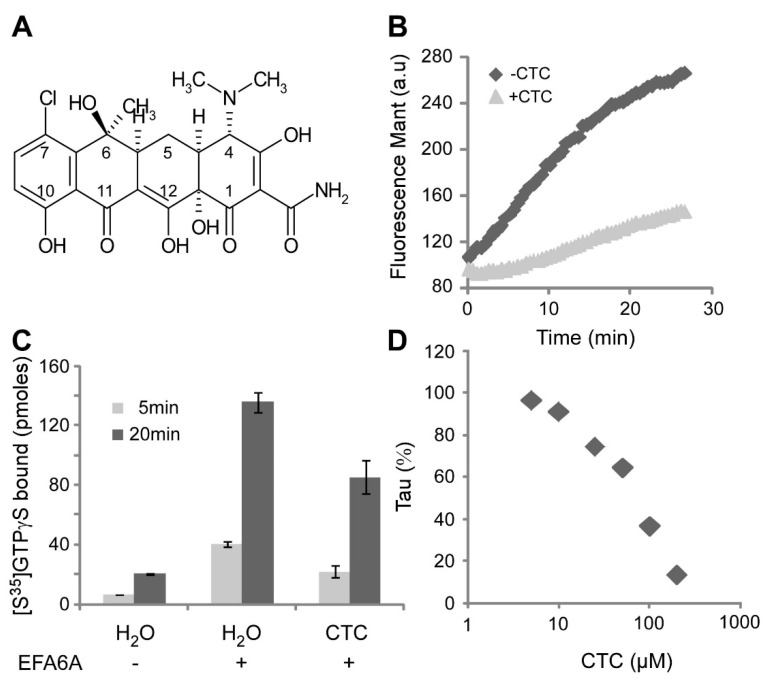
CTC blocks Arf6 GTP loading in vitro. (**A**) Chemical structure of chlortetracycline (CTC). (**B**) mant-GTP loading on Arf6gly (2 µM) was carried out in presence of EFA6 (0.2 µM) and azolectin (0.4 g/L), with or without 100 µM CTC. Normalized fluorescence of a representative experiment is shown. (**C**) GTPγS^35^ loading on Arf6 was done with or without EFA6A and CTC for 5 and 20 min. (**D**) The mant-GTP loading experiment was done for different CTC concentrations and the slope of the resulting curves (tau in ∆fluorescence per seconds) were measured and normalized without drug. IC50 was calculated at 75 µM.

To search for molecules that inhibit Arf6, we developed a high throughput ad hoc in vitro assay based on Arf6 activation stimulated by EFA6A. The guanine nucleotide exchange of the full-length Arf6 was carried out in the presence of liposomes to faithfully mimic the in vivo enzymatic reaction, which includes the toggle of the *N*-terminal amphiphatic helix and its binding to the lipid membrane during GTP binding-induced conformational change. The nucleotide exchange reaction was followed by fluorescence resonance energy transfer (FRET) between an Arf6 tryptophan and the fluorescent mant (2’/3’-*O*-(*N*-Methyl-anthraniloyl)-labelled GTP (mant-GTP) bound to Arf6. In total, 1120 FDA-approved compounds from the Prestwick library were screened at a concentration of 100 µM. Six molecules inhibited the EFA6A stimulated activation of Arf6. We focused our study on a cell-permeant compound, chlortetracyclin (CTC), which was the most effective among several molecules of the same family (Figure 1A,B).

To exclude the possibility that the inhibitory activity of CTC was associated with the mant-labeled nucleotide, we carried out an assay based on radioactive GTP-binding. We observed in the presence of EFA6A a strong increase of [S^35^]GTPγS binding to Arf6, which was significantly reduced in the presence of the drug CTC, in the same proportion as observed by fluorescence (Figure 1C). In addition, we performed a dose-response using a distinct set of preparations of EFA6A and Arf6 and a new batch of CTC purchased from another source (Figure 1D). We observed a dose-dependent inhibition with an IC50 of 75 µM.

**Figure 2:**
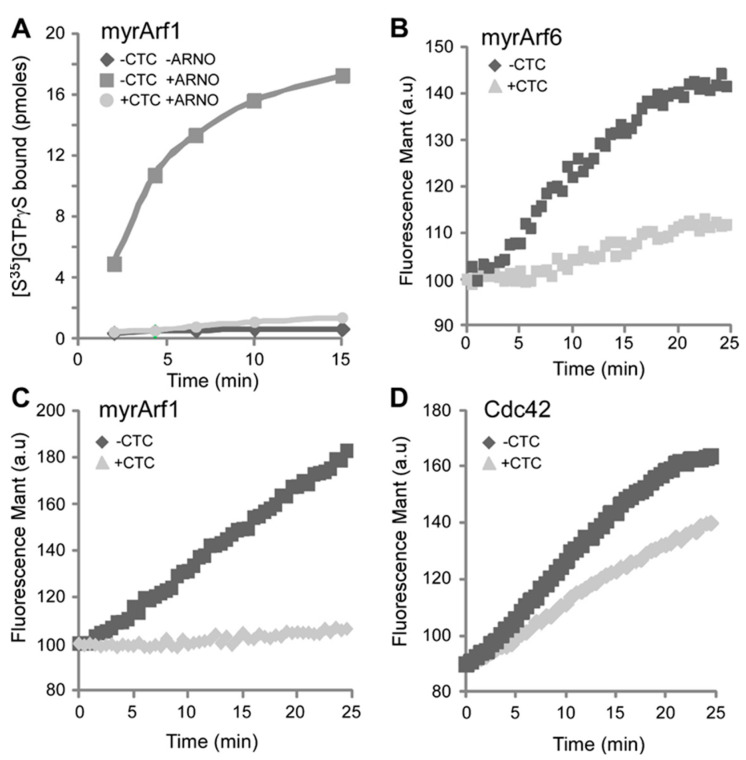
CTC is a specific inhibitor of Arf GTP loading in vitro. (**A**) GTPγS ^35^ (10 µM) loading on myrArf1 (2 µM) was measured at 22 °C in the presence of 0.4 g/L azolectin with or without Arno (4 nM) and CTC (100 µM). (**B)** mant-GTP (5 µM) loading on myrArf6 (2.5 µM) was done at 37 °C in the presence of 0.4 g/L azolectin with or without CTC (100 µM). (**C**) mant-GTP (5 µM) loading on myrArf1 (2.5 µM) was done at 37 °C in the presence of 0.4 g/L azolectin with or without CTC (100 µM). (**D**) mant-GTP (5 µM) loading on Cdc42 (2 µM) was done at 22 °C in the presence of 0.4 g/L azolectin with or without CTC (100 µM).

Next, we assessed the specificity of the drug on Arf1 that shares 67% identity with Arf6 and a very similar structure (r.m.s.d. of 0.631 Å). The assay was carried out with its specific GEF ARNO. We observed that CTC also inhibited the ARNO-catalyzed exchange of mant-GTP on the myristoylated form of Arf1 (myrArf1) (Figure 2A). Because the drug could inhibit GTP exchange on both EFA6A/Arf6 and ARNO/Arf1, we suspected that CTC could affect the spontaneous nucleotide exchange independently of the presence of the exchange factors. Indeed, CTC was able to strongly inhibit the spontaneous activation on the myristoylated forms of Arf6 (myrArf6) and Arf1 (myrArf1) (Figure 2B,C). We then examined the specificity of the compound towards a small G protein from a distinct family. We measured the effect on the spontaneous nucleotide exchange by Cdc42 (Cell Division Cycle 42) and found only a mild effect of the drug (Figure 2D). We conclude that CTC selectively affects the activity of the Arf family.

### 2.2. CTC Does Not Compete with the Nucleotide for Binding to Arf6 

**Figure 3:**
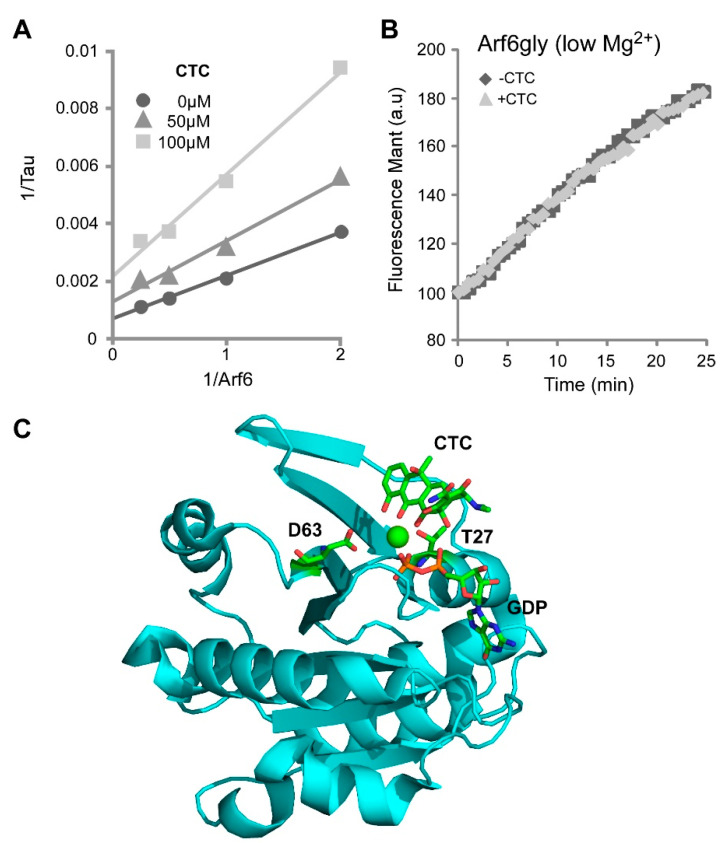
CTC is a non-competitive inhibitor of Arf GTP loading in vitro. (**A**) Arf6 mant-GTP loading was measured with different CTC and Arf6 concentrations. A Lineweaver–Burk plot is represented. (**B**) Arf6 mant-GTP loading was done with 2 mM EDTA (1 µM free MgCl_2_) with or without CTC (100 µM). (**C**) Representation of the 3D modeling of the CTC-Arf6-MgGDP complex.

To determine the mode of action of CTC, the inhibition assay was repeated by varying the concentrations of drug and Arf6 in the presence of constant amounts of EFA6A and mant-GTP (Figure 3A). Since the Vmax varied while the Km stayed constant, we deduced that the drug CTC acts as a non-competitive inhibitor, indicating that CTC binds Arf6 to a distinct allosteric site from that of the nucleotide.

Tetracycline derivatives have been shown to bind magnesium ions with a ~3 mM affinity [19]. Interestingly, Mg^2+^ is present in the guanine nucleotide site of G proteins where it plays a crucial role in the binding of the nucleotide. Therefore, we asked whether magnesium is required for the binding of CTC to Arf6 and for its inhibition effect. At low magnesium concentration (~1 µM free Mg^2+^), a condition in which the nucleotide exchange is favored, we observed that the drug had no effect on Arf6 activation (Figure 3B). This result suggested that CTC inhibitory activity required the presence of a magnesium ion.

Altogether our data indicate that CTC is a non-competitive inhibitor that interacts with the Mg^2+^ present in the nucleotide binding site and inhibits the GDP/GTP exchange. We used the crystallographic structure of tetracycline in complex with *Escherichia coli* EF-Tu-MgGDP [20] as a model to visualize CTC and Arf6-MgGDP binding. Similarly to this model, our computing simulation of the 3D structure of CTC-Arf6-MgGDP complex predicts a direct association of the phenol diketone part of CTC with the Mg^2+^, the nucleotide and two amino acids, T27 and D63, of Arf6 (Figure 3C). T27 is a conserved residue in the P-loop that interacts with the β-phosphate and the magnesium ion, whereas D63 is localized in the conserved DVGGQ motif of the switch II domain, also involved in nucleotide binding. Thus, this predicted model of CTC binding to Arf6 is entirely consistent with our in vitro experimental results, i.e., a non-competitive association, a Mg^2+^ dependency and a role in nucleotide interaction.

Altogether, our experiments show that CTC interacts with the small G protein Arf and inhibits both its catalyzed and spontaneous activation.

### 2.3. CTC Inhibits the GTP-Dependent Conformational Change That Leads to the Binding of Effectors

**Figure 4:**
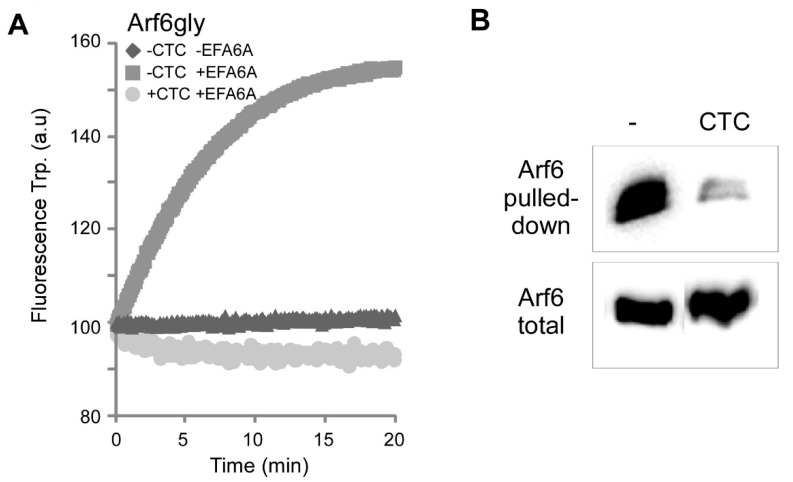
CTC blocks the Arf6GTP-induced change of conformation. (**A**) GTP (250 µM) loading on Arf6gly (1 µM) was monitored by tryptophan fluorescence (Ex: 297 nM, Em: 340 nM) at 25 °C with 0.2 g/L azolectin and with or without EFA6A (250 nM) and CTC (100 µM). (**B**) Arf6GTP binding to ARHGAP10 was carried out by a GST pull-down experiment as described in Methods.

In order for the drug to be useable in vivo, it needs not only to block the nucleotide exchange but foremost to prevent the conformational change that comes along. To verify that CTC inhibits the conformational activated state of Arf6, that is the switch toggle that exposes the effector binding domain, we performed two additional assays. First, we measured the change of the intrinsic fluorescent of tryptophan residues in Arf6 that reflects the conformational change occurring upon GTP binding. In the presence of EFA6A, CTC effectively prevented the fluorescence increase (Figure 4A). Second, we performed a pull-down experiment with the purified Arf-binding domain of the Arf effector GGA3. In the presence of GTP and absence of CTC (control condition), Arf6 was efficiently pulled-down by GST-GGA3. In contrast, the addition of CTC totally abolished the binding of Arf6 to its effector domain (Figure 4B). These results clearly indicate that CTC inhibits the conversion of Arf6 towards its active state and consequently its capability to bind its effectors.

### 2.4. CTC Inhibits the Serum-Induced Activation of Arf6 In Vivo

**Figure 5:**
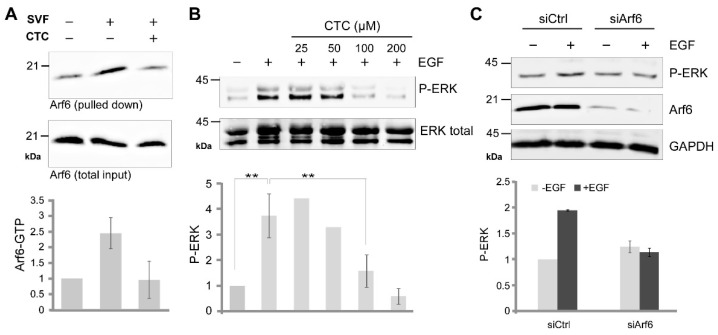
CTC blocks EGF-induced MAPK phosphorylation. (**A**) Serum-induced Arf6GTP loading was measured in starved RPE-1 cells with or without treatment with CTC (100 µM) for 2 h (n = 3). The amount of Arf6GTP was determined as described in experimental procedures. Arf6 western blot was quantified on the bottom panel. (**B**) After starvation, RPE-1 cells were pre-treated with different CTC concentrations for 2 h and activated or not with EGF (50 ng/mL) for 15 min. P-ERK western blot was quantified on the bottom panel (n = 3, 0.001 < ** *p* < 0.01). (**C**) RPE-1 cells were transfected with non-relevant siRNA (siCtrl) or anti-Arf6 siRNA (siArf6) for 3 days and stimulated or not with EGF (50 ng/mL). P-ERK western blot was quantified on the bottom panel (n = 3).

Since CTC is cell permeant, we assessed whether it could inhibit the activation of Arf6 in cells. As previously described, addition of serum to fibroblast or epithelial cells triggers the activation of Arf6 [4,21]. Indeed, we observed that the addition of 5% of serum for 15 min to RPE-1 cells after 4 h starvation caused an increase of Arf6GTP pulled-down by GST-GGA3 (Figure 5A). In contrast, no accumulation of activated Arf6 was observed in the presence of CTC at 150 µM. Thus, our results indicate that CTC inhibits the serum-stimulated activation of Arf6 in cells.

### 2.5. CTC Inhibits the Arf6-Dependent Activation of the MAP Kinase Pathway In Vivo

EGF was reported to stimulate the Arf6-dependent activation of the MAPK cascade [22]. Addition of EGF (50 ng/mL) to serum-starved RPE-1 cells triggers the phosphorylation of ERK, which is inhibited in a dose-dependent manner by CTC (Figure 5B), similarly to siRNA-mediated depletion of Arf6 (Figure 5C). Thus, CTC can inhibit Arf6 activation and an Arf6-associated signaling pathway in RPE-1 cells.

### 2.6. CTC Inhibits Serum and EGF-Stimulated Migration of RPE-1 Cells

**Figure 6:**
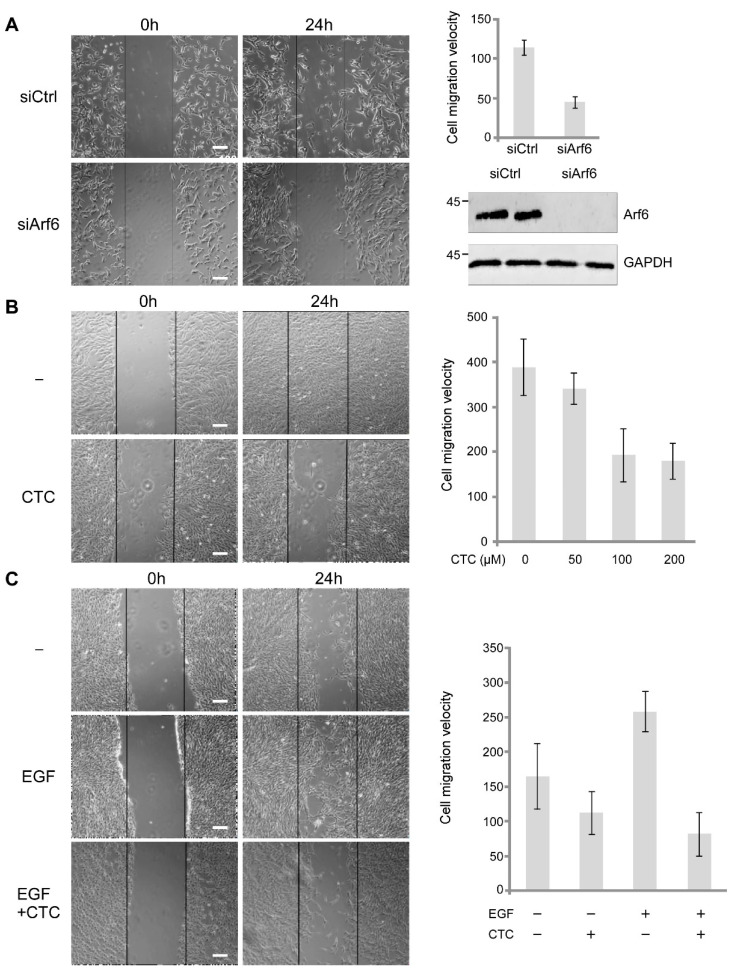
CTC blocks EGF-induced RPE cells migration. (**A**) Cells were transfected with non-relevant siRNA (siCtrl) or anti-Arf6 siRNA (siArf6) for 3 days and a wound healing experiment was performed. Speed of wound closure was determined using ImageJ software. (**B**) Anti-Arf6 western blot was performed to control siRNA inactivation. (**C**) As indicated, several CTC concentrations were used in the wound healing assay.

Arf6 participates in the regulation of the motility of various cell lines [19,20,21]. Using a wound-healing assay, we examined the impact of CTC on the migratory properties of RPE-1 cells. We first determined that the migration of RPE-1 cells was Arf6-dependent in the presence of serum. Indeed, we observed a two-fold reduction of the migration rate of RPE-1 cells upon Arf6 depletion (Figure 6A).

Next, we found that CTC reduced the speed of wound closure in a dose-dependent manner to the same extent as Arf6 depletion (Figure 6B). We also tested the effect of CTC on the EGF-stimulated cell motility in the absence of serum. First, we found that the drug was able to slightly slow down the migration rate of unstimulated cells in the absence of serum. More interestingly, CTC totally abolished the two-fold stimulation of migration induced by EGF (Figure 6C). Altogether, our results show that CTC inhibits the Arf6-dependent cell migration.

### 2.7. CTC Inhibits the Collective Invasive Properties of MBA-MB-231 Cells in Collagen

**Figure 7:**
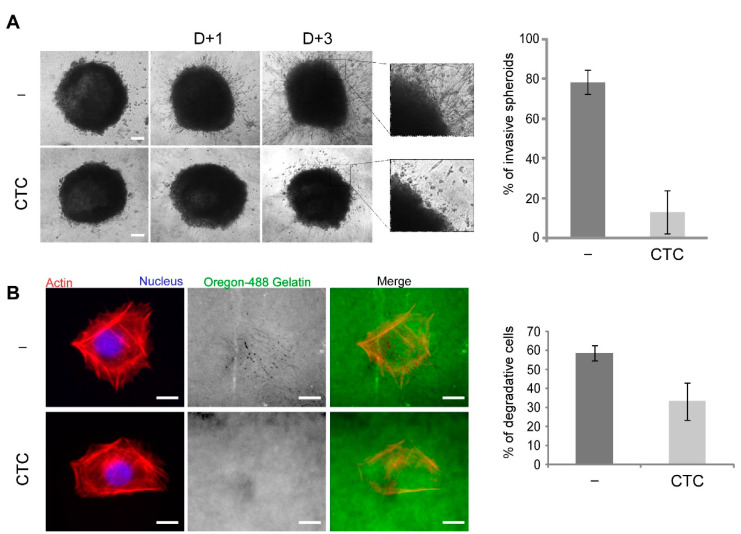
CTC inhibits MDA-MB-231 cell invasion and matrix degradation properties. (**A**) Spheroid formation of MDA-MB-231 cells in collagen was monitored for 3 days with or without CTC (100 µM). The graph (right panel) represents the % of invasive spheroids estimated after 3 days (n = 100 per experiment) compared to t = 0 from 3 independent experiments performed in quadruplicates. The mean is represented ± SD. Scale bar 100 µm. (**B**) Representative images of MDA-MB-231 placed in Oregon-488 gelatin (green) coated coverslips and stained for F-actin (red) and nuclei (blue). Areas devoid of fluorescent signal indicate degradation of the fluorescent gelatin. Scale bars 10 μm. Quantification of degrading cells (right panel). Values are percentages of degrading cells, in which 100 cells per condition were analyzed. n = 3, Student’s *t*-test, * *p* < 0.05.

The role of Arf6 in tumor progression is well established in breast cancer models. We thus asked whether CTC could inhibit the invasive properties of the invasive breast cancer cell line MDA-MB-231. Spheroids placed in 2 mg/mL collagen I in the presence or absence of CTC were imaged at regular times by phase contrast microscopy. After one day, MDA-MB-231 cells could be observed migrating away from the spheroid and after 3 days, single cells and cell strands were seen extensively invading the collagen matrix (Figure 7A). In contrast, when CTC was added in the culture medium, invasion was strongly reduced. Quantification of the percentage of invasive spheroids revealed an 8-fold decrease (Figure 7A). Under these conditions, invasion relies on the degradation of the collagen matrix by metalloproteases [23]. We thus asked whether CTC could inhibit the degradative properties of MDA-MB-231 cells. We carried out a gelatin-degradation assay. In the absence of the drug, 58% of cells were found to degrade the fluorescent gelatin, while only 33% did so in the presence of CTC (Figure 7B). We conclude that CTC inhibits both the migratory and degradative properties of MDA-MB-231 cells accounting for the anti-invasive effect of CTC.

## 3. Discussion

Arf6 and its regulators, arguably involved in cancer progression, are promising drug targets for cancer therapy [17,22]. To search for Arf6 inhibitors, we set up a miniaturized in vitro screening assay that recapitulates GEF-stimulated Arf6 nucleotide exchange at the surface of lipid membranes. The high cost and low success rates of very large chemical library (containing biochemically and biologically uncharacterized compounds) high-throughput screenings balanced with the recent discoveries for new applications of “old” drugs make repurposing attractive. Therefore, we screened a small chemical library of already FDA-approved compounds. The availability of the compound information, such as its biological activity, pharmacokinetics and toxicity should render our process of drug development far more efficient.

Interestingly, we found that CTC a well-known antibiotic compound is able to strongly inhibit the small G protein Arf6 activation both in vitro and in vivo. CTC is a member of the large tetracyclin family that binds to the 30S ribosomal subunit and to the bacterial G protein elongation factor EF-Tu. It is tempting to think that CTC interacts with Arf6 in a similar way as tetracyclins bind to the bacterial G protein elongation factor EF-Tu. Indeed, we found that CTC is a non-competitive Arf6 inhibitor, indicating that its binding site on Arf6 is distinct to that of the nucleotide, similarly to the tetracyclin in its complex with EF-Tu-GDP [24]. Moreover, as it has been shown for tetracyclin [24,25], we observed that CTC interacts with the magnesium ion present in most G proteins to stabilize the nucleotide binding. Importantly, it should be noted that among the dozen tetracyclin members we tested, CTC was the most efficient, indicating that Arf6 specifically binds to CTC, making it a promising drug.

**Figure A1:**
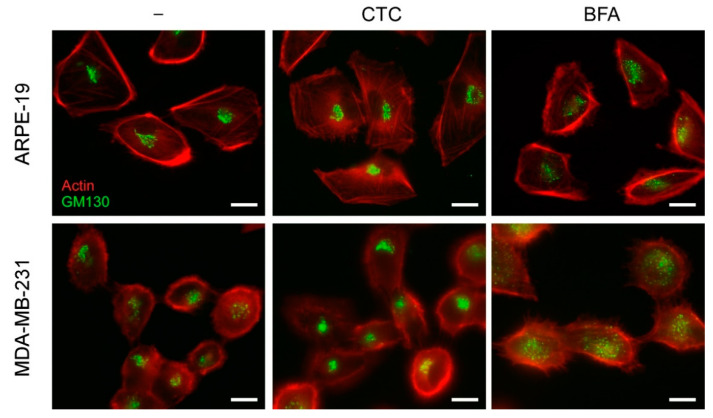
CTC does not affect Golgi morphology. ARPE-19 and MDA-MB-231 cells were treated with CTC at 100 μM and BFA at 10 μg/mL. ARPE-19 and MDA-MB-231 cells were treated for 2 h and 45 min, respectively. Golgi morphology immunofluorescent staining was performed using anti-GM130 antibody. Representative images of cells stained for the cis-Golgi GM130 (green) and F-actin markers (Phalloidin; red). Scale bar: 10 μM.

Due to their high sequence and structure homologies, it was not surprising that in vitro CTC could efficiently inhibit Arf6 and Arf1. However, in cells and in contrast to BFA, CTC does not disturb the morphology of the Golgi, a major Arf1-regulated function (Figure A1). A likely explanation is that Arf1-associated Golgi activity is strictly dependent upon its activation by large Arf1-GEFs that might be insensitive to CTC. This is reminiscent of the aptamer M69 that inhibits Arf1 catalyzed nucleotide exchange by cytohesin-1 and cytohesin-2 but not by the large Arf-GEF Gea2 [26]. The chemical Arf1 inhibitor secinH3 also inhibits cytohesin-associated functions but does not disrupt the Golgi [23]. Another possibility would be that, in vivo, CTC for some unknown reason, specifically inhibits Arf6. In any case, the absence of a deleterious impact on the Golgi apparatus is a great advantage in the perspective of developing an anti-tumor drug. In addition, CTC does not affect the spontaneous nucleotide exchange of Cdc42, indicating a specificity for the Arf family members. 

Since the discovery of the BFA, a few additional drugs targeting large and small Arf-GEFs have been reported [17], the mode of action of which has not always been clearly characterized. Our biochemical characterization demonstrates that CTC affects the spontaneous and the stimulated exchange of nucleotides by non-competitive inhibition. The corollary is that the inhibition of Arf6 is effective regardless of the Arf6-GEF. Although this may appear to generate a non-selective inhibitory effect of all Arf6-regulated pathways, we believe that Arf6 is likely to play pleiotropic roles to promote invasion through MMP14 cell surface transport, invadopodia formation, migration, growth factor trafficking, the Warburg effect, etc. [27]. A drug that targets all Arf6 pathways rather than one that would be selective for a particular Arf-GEF might prove more efficient in vivo.

In cells, we found that CTC blocks the activation of Arf6 upon exposure to two stimuli, serum and EGF, and the Arf6-regulated ERK pathway and inhibits two known Arf6-dependent cell functions, migration and invasion in 3D collagen I. Interestingly, members of the tetracyclin family have been found to inhibit matrix metalloproteinases [28]. Tetracyclines may act at two levels: first they inhibit the enzymatic activity, probably by chelating Zn^2+^ required for protease activity, and second they could inhibit MMP expression levels (reviewed in [29]). As Arf6 is required for the formation of invadopodia and for the targeting of MMPs from intracellular compartments to the plasma membrane (reviewed in [7]), we hypothesize that by inhibiting Arf6 activation, CTC would inhibit the invasive properties of cancer cells. Thus, CTC could block cellular invasion by inhibiting both the Arf6-mediated invadopodia formation and MMPs.

The acquisition of invasive properties determines the malignancy of a tumor, and metastasis is the major cause of cancer-associated mortality, yet today, no anti-invasion treatment is available. Here, we report the first characterization of a well-known antibiotic drug targeting Arf6-associated functions that endow tumor cells with disseminating properties.

Further studies will be undertaken in order to improve the affinity of this molecule for Arf6 and test its anti-metastatic properties in orthotopic mammary MDA-MB-231 tumors developed in mice. 

## 4. Materials and Methods

### 4.1. Expression and Purification of Recombinant Proteins 

For the in vitro assays, recombinant myristoylated Arf6 and Arf1 (myrArf6, myrArf1) and non myristoylated Arf6 (Arf6) with a C-terminal hexa-his tag and recombinant his-tagged EFA6A (His-EFA6A) and ARNO (His-ARNO) were prepared as previously described [30]. GST-Cdc42 was prepared as previously described [31].

### 4.2. Preparation of Phospholipid Vesicles

Large unilamellar vesicles of azolectin were prepared as previously described [32], and extruded through a 0.4 µM pore size polycarbonate filter (Isopore, Millipore, Molsheim France). 

### 4.3. Mant-GTP Binding Assay

Arf6-His (2 µM), myrArf1 (2 µM), myrArf6 (2 µM) with or without EFA6A (0.2 µM) or Arno (4 nM) and azolectin (0.4 g/L) or Cdc42-His alone were incubated in the presence of 5 µM mantGTP in 20 mM Hepes pH 7.4, 100 mM KCl, 1 mM MgCl_2_. Fluorescence (Ex: 275 nM, Em 440 nM) was taken every 30 s during the kinetics (TECAN infinite M1000Pro).

### 4.4. Prestwick Chemical Library Screening

The screen was done as indicated in the mant-GTP binding assay in a final volume of 50 µL. Sample handling was done using Biotek precision 2000 automated pipetting system. Drugs were obtained from the Prestwick Chemical Library (Prestwick Chemical Inc., Illkirch-Graffenstaden, France) and used at 100 µM. 

### 4.5. [^35^S]GTPγS Binding Assay

MyrArf1 (2 µM) was incubated at 22 °C with [S^35^]GTPγS (20 µM, ~ 2000 cpm/pmol) in 50 mM Hepes (pH 7.5), 2 mM MgCl_2_, 100 mM KCl, with azolectin (2 mM) with or without (as indicated in the figure legend) His-tagged EFA6A (0.5 µM). At the indicated times, samples of 25 µL were removed and measured for radioactivity as described previously [33].

### 4.6. GST Pull-Down Experiments

Cells were lysed at 4 °C in 0.5% Nonidet P-40, 20 mM Hepes pH 7.4, 125 mM NaCl, 1 mM phenylmethylsulphonyl fluoride (PMSF) and a cocktail of protease inhibitors (Complete, Roche Diagnostics GmbH, Mannheim Germany). The cleared lysates containing the protein of interest were incubated for 4 h at 4 °C with 1.5 μM of the GST or GST fused to the Arf6GTP binding domain of ARHGAP10 proteins and 30 μL of glutathione-sepharose CL-4B beads (GE Healthcare). After three washes in lysis buffer, the beads were boiled in Laemmli buffer, and submitted to SDS–PAGE followed by immunoblot. The proteins were revealed by chemiluminescence (GE Healthcare) and the membranes analyzed with the luminescent analyzer LAS-3000 (Fujifilm).

### 4.7. Cell Culture 

RPE-1, ARPE-19 and MDA-MB-231 cells were obtained from and grown according to ATCC instructions. All culture reagents were from Invitrogen (Fisher Scientific, Illkirch France) except for the fetal calf serum (Dutscher, Bernolsheim France). For serum starvation, cells were washed 3 times in PBS and incubated in culture medium without serum for the indicated times. 

### 4.8. Antibodies and Reagents 

Rabbit polyclonal antibodies specific for GAPDH (Sigma-Aldrich, St. Quentin Fallavier, France) and ERK (Millipore), rabbit monoclonal antibodies specific for phosphor-ERK (Cell Signaling Technology, Leiden, Netherlands), and mouse monoclonal antibodies specific for GM130 (BD Transduction Laboratories) were used. The mouse monoclonal anti-Arf6 was described elsewhere [34]. All secondary antibodies and fluorescent probes were from Molecular Probes (Invitrogen, Cergy Pontoise, France). Unless otherwise indicated, all reagents were from (Sigma-Aldrich, France).

### 4.9. siRNAs and Mammalian Expression Constructs 

siRNA transfection in RPE-1 and MDA-MB-231 cells was performed using RNAiMAX (Invitrogen) following the manufacturer’s instructions and the transfected cells were incubated for 3 days at 37 °C. siRNA targeting human Arf6, #a:5’-CGGCAUUACUACACUGGGA-3’; #b:5’-ACGUGGAGACGGUGACUUA-3’ (Sigma-Aldrich) was used.

### 4.10. Wound-Healing

RPE-1 cells were plated confluent in a 12-well dish (106 cells per well). The next day a wound was performed using a pipet tip. After a wash with complete medium, the closure of the wound was followed overtime by phase contrast video-microscopy using the Cytation 5 imaging system (Biotek, Colmar, France). Images were taken every 15 min and the area of the wound closure calculated using the software ImageJ. The graph represents the percentage of closure relative to the surface area of the wound at t = 0 from 3 independent experiments performed in triplicates ± SD.

### 4.11. Collagen I Invasion Assay 

MDA-MB-231 spheroids were prepared by making 20 µL hanging-drops containing 40 × 10^3^ cells in complete medium supplemented with 0.5 mg/mL Matrigel. After 24 h, a solution of 2.5 mg/mL collagen I was prepared with or without CTC at 125 µM. The collagen I solution (80 µL) was mixed with the 20 µL hanging-drop containing spheroids and plated in a 96 well-plate. Phase contrast images were recorded and the percentage of invasive spheroids was assessed at day 3 by blind evaluation by two independent readers. 

### 4.12. Fluorescent Gelatin Degradation Assay

Oregon Green 488-labeled gelatin was from Molecular Probes (Invitrogen). Sterilized coverslips (18-mm diameter) were coated with 50 μg/mL poly-L-lysine for 20 min at RT, washed with PBS (phosphate buffered saline), and fixed with 0.5% glutaraldehyde for 10 min. After 3 washes with PBS, the coverslips were inverted on a 40-μL drop of 0.2% fluorescently labeled gelatin in 2% sucrose in PBS and incubated for 10 min at RT. After washing with PBS, coverslips were incubated in a 5 mg/mL solution of borohydride for 3 min, washed three times in PBS, and incubated with 1 mL of complete medium for 30 min. 5 × 10^4^ cells per 12-well were plated on the fluorescent gelatin-coated coverslips and incubated at 37 °C for 48 h. Cells were then washed three times with PBS and fixed with 4% PFA (paraformaldehyde) for 20 min and processed for labeling with Texas Red-Phalloidin and DAPI (diamino-2-phenylindole). The coverslips were mounted with Mowiol mounting medium. Cells were imaged on a confocal microscope (Leica TCS-SP5). The graph represents the percentage of degradative cells (n = 100 per experiment) compared to t = 0 of 3 independent experiments ± SD. 

### 4.13. Quantifications

Results are the mean of three to five independent experiments. The error bars represent standard deviations within n experiments (n = number of distinct experiments, unless otherwise indicated). Statistical significances (p) were calculated using the Student’s test. * *p* < 0.05, ** *p* < 0.01, *** *p* < 0.001 and **** *p* < 0.0001.

## Data Availability

All data supporting the presented study are available upon request to the corresponding author.

## References

[1] Hashimoto S., Onodera Y., Hashimoto A., Tanaka M., Hamaguchi M., Yamada A., Sabe H. (2004). Requirement for Arf6 in breast cancer invasive activities. Proc. Natl. Acad. Sci. USA.

[2] Li M., Ng S.S., Wang J., Lai L., Leung S.Y., Franco M., Peng Y., He M.L., Kung H.F., Lin M.C. (2006). EFA6A enhances glioma cell invasion through ADP ribosylation factor 6/extracellular signal-regulated kinase signaling. Cancer Res..

[3] Morishige M., Hashimoto S., Ogawa E., Toda Y., Kotani H., Hirose M., Wei S., Hashimoto A., Yamada A., Yano H. (2008). GEP100 links epidermal growth factor receptor signalling to Arf6 activation to induce breast cancer invasion. Nat. Cell Biol..

[4] Tague S.E., Muralidharan V., D’Souza-Schorey C. (2004). ADP-ribosylation factor 6 regulates tumor cell invasion through the activation of the MEK/ERK signaling pathway. Proc. Natl. Acad. Sci. USA.

[5] Grossmann A.H., Yoo J.H., Clancy J., Sorensen L.K., Sedgwick A., Tong Z., Ostanin K., Rogers A., Grossmann K.F., Tripp S.R. (2013). The small GTPase ARF6 stimulates beta-catenin transcriptional activity during WNT5A-mediated melanoma invasion and metastasis. Sci. Signal..

[6] Hu B., Shi B., Jarzynka M.J., Yiin J.J., D’Souza-Schorey C., Cheng S.Y. (2009). ADP-ribosylation factor 6 regulates glioma cell invasion through the IQ-domain GTPase-activating protein 1-Rac1-mediated pathway. Cancer Res..

[7] Li R., Peng C., Zhang X., Wu Y., Pan S., Xiao Y. (2017). Roles of Arf6 in cancer cell invasion, metastasis and proliferation. Life Sci..

[8] Oka S., Uramoto H., Shimokawa H., Yamada S., Tanaka F. (2014). Epidermal growth factor receptor-GEP100-Arf6 axis affects the prognosis of lung adenocarcinoma. Oncology.

[9] Marchesin V., Castro-Castro A., Lodillinsky C., Castagnino A., Cyrta J., Bonsang-Kitzis H., Fuhrmann L., Irondelle M., Infante E., Montagnac G. (2015). ARF6-JIP3/4 regulate endosomal tubules for MT1-MMP exocytosis in cancer invasion. J. Cell Biol..

[10] Davis J.E., Xie X., Guo J., Huang W., Chu W.M., Huang S., Teng Y., Wu G. (2016). ARF1 promotes prostate tumorigenesis via targeting oncogenic MAPK signaling. Oncotarget.

[11] Sasamori T., Takeda N., Fujio M., Kimura M., Nagase S., Tokitoh N. (2002). Synthesis and structure of the first stable phosphabismuthene. Angew. Chem. Int. Ed. Engl..

[12] Schlienger S., Ramirez R.A., Claing A. (2015). ARF1 regulates adhesion of MDA-MB-231 invasive breast cancer cells through formation of focal adhesions. Cell Signal..

[13] Xu X., Wang Q., He Y., Ding L., Zhong F., Ou Y., Shen Y., Liu H., He S. (2017). ADP-ribosylation factor 1 (ARF1) takes part in cell proliferation and cell adhesion-mediated drug resistance (CAM-DR). Ann. Hematol..

[14] Gu G., Chen Y., Duan C., Zhou L., Chen C., Chen J., Cheng J., Shi N., Jin Y., Xi Q. (2017). Overexpression of ARF1 is associated with cell proliferation and migration through PI3K signal pathway in ovarian cancer. Oncol. Rep..

[15] Zhou L., Gao W., Wang K., Huang Z., Zhang L., Zhang Z., Zhou J., Nice E.C., Huang C. (2019). Brefeldin A inhibits colorectal cancer growth by triggering Bip/Akt-regulated autophagy. FASEB J..

[16] Benabdi S., Peurois F., Nawrotek A., Chikireddy J., Caneque T., Yamori T., Shiina I., Ohashi Y., Dan S., Rodriguez R. (2017). Family-wide Analysis of the Inhibition of Arf Guanine Nucleotide Exchange Factors with Small Molecules: Evidence of Unique Inhibitory Profiles. Biochemistry.

[17] Bourgoin S., El Azeq M.-A. (2012). Small inhibitors of ADP-ribosylation factors activation and function in mammalian cells. World J. Pharmacol..

[18] Gzyl K.E., Wieden H.J. (2017). Tetracycline does not directly inhibit the function of bacterial elongation factor Tu. PLoS ONE..

[19] Palacios F., Price L., Schweitzer J., Collard J.G., D’Souza-Schorey C. (2001). An essential role for ARF6-regulated membrane traffic in adherens junction turnover and epithelial cell migration. Embo J..

[20] Santy L.C., Casanova J.E. (2001). Activation of ARF6 by ARNO stimulates epithelial cell migration through downstream activation of both Rac1 and phospholipase D. J. Cell Biol..

[21] Svensson H.G., West M.A., Mollahan P., Prescott A.R., Zaru R., Watts C. (2008). A role for ARF6 in dendritic cell podosome formation and migration. Eur. J. Immunol..

[22] Vigil D., Cherfils J., Rossman K.L., Der C.J. (2010). Ras superfamily GEFs and GAPs: Validated and tractable targets for cancer therapy?. Nat. Rev. Cancer.

[23] Hafner M., Schmitz A., Grune I., Srivatsan S.G., Paul B., Kolanus W., Quast T., Kremmer E., Bauer I., Famulok M. (2006). Inhibition of cytohesins by SecinH3 leads to hepatic insulin resistance. Nature.

[24] Heffron S.E., Mui S., Aorora A., Abel K., Bergmann E., Jurnak F. (2006). Molecular complementarity between tetracycline and the GTPase active site of elongation factor Tu. Acta Crystallogr. D Biol. Crystallogr..

[25] Aleksandrov A., Simonson T. (2008). Binding of tetracyclines to elongation factor Tu, the Tet repressor, and the ribosome: A molecular dynamics simulation study. Biochemistry.

[26] Mayer G., Blind M., Nagel W., Bohm T., Knorr T., Jackson C.L., Kolanus W., Famulok M. (2001). Controlling small guanine-nucleotide-exchange factor function through cytoplasmic RNA intramers. Proc. Natl. Acad. Sci. USA.

[27] Castro-Castro A., Marchesin V., Monteiro P., Lodillinsky C., Rosse C., Chavrier P. (2016). Cellular and Molecular Mechanisms of MT1-MMP-Dependent Cancer Cell Invasion. Annu. Rev. Cell Dev. Biol..

[28] Golub L.M., Lee H.M., Ryan M.E., Giannobile W.V., Payne J., Sorsa T. (1998). Tetracyclines inhibit connective tissue breakdown by multiple non-antimicrobial mechanisms. Adv. Dent. Res..

[29] Castro M.M., Kandasamy A.D., Youssef N., Schulz R. (2011). Matrix metalloproteinase inhibitor properties of tetracyclines: Therapeutic potential in cardiovascular diseases. Pharmacol. Res..

[30] Boulakirba S., Macia E., Partisani M., Lacas-Gervais S., Brau F., Luton F., Franco M. (2014). Arf6 exchange factor EFA6 and endophilin directly interact at the plasma membrane to control clathrin-mediated endocytosis. Proc. Natl. Acad. Sci. USA.

[31] Dubois T., Paleotti O., Mironov A.A., Fraisier V., Stradal T.E., De Matteis M.A., Franco M., Chavrier P. (2005). Golgi-localized GAP for Cdc42 functions downstream of ARF1 to control Arp2/3 complex and F-actin dynamics. Nat. Cell Biol..

[32] Franco M., Chardin P., Chabre M., Paris S. (1995). Myristoylation of ADP-ribosylation factor 1 facilitates nucleotide exchange at physiological Mg2+ levels. J. Biol. Chem..

[33] Franco M., Peters P.J., Boretto J., van Donselaar E., Neri A., D’Souza-Schorey C., Chavrier P. (1999). EFA6, a sec7 domain-containing exchange factor for ARF6, coordinates membrane recycling and actin cytoskeleton organization. Embo J..

[34] Marshansky V., Bourgoin S., Londono I., Bendayan M., Vinay P. (1997). Identification of ADP-ribosylation factor-6 in brush-border membrane and early endosomes of human kidney proximal tubules. Electrophoresis.

